# The role of IGF-1 in exercise to improve obesity-related cognitive dysfunction

**DOI:** 10.3389/fnins.2023.1229165

**Published:** 2023-08-10

**Authors:** Yimin He, Qian Wang, Huaduo Wu, Yumo Dong, Zifu Peng, Xiangyin Guo, Ning Jiang

**Affiliations:** Tianjin Key Laboratory of Exercise Physiology and Sports Medicine, Institute of Sport, Exercise and Health, Tianjin University of Sport, Tianjin, China

**Keywords:** obesity, IGF-1, cognitive function, neurogenesis, synaptic plasticity, oxidative stress, inflammation, exercise

## Abstract

Obesity is an important factor that threatens human health. The occurrence of many chronic diseases is related to obesity, and cognitive function decline often occurs with the onset of obesity. With the further prevalence of obesity, it is bound to lead to a wider range of cognitive dysfunction (ORCD). Therefore, it is crucial to suppress ORCD through intervention. In this regard, exercise has been shown to be effective in preventing obesity and improving cognitive function as a non-drug treatment. There is sufficient evidence that exercise has a regulatory effect on a growth factor closely related to cognitive function—insulin-like growth factor 1 (IGF-1). IGF-1 may be an important mediator in improving ORCD through exercise. This article reviews the effects of obesity and IGF-1 on cognitive function and the regulation of exercise on IGF-1. It analyzes the mechanism by which exercise can improve ORCD by regulating IGF-1. Overall, this review provides evidence from relevant animal studies and human studies, showing that exercise plays a role in improving ORCD. It emphasizes the importance of IGF-1, which helps to understand the health effects of exercise and promotes research on the treatment of ORCD.

## 1. Introduction

Obesity is a state in which the body consumes too many calories but consumes less, resulting in abnormal or excessive accumulation of adipose tissue (World Health Organization, 2000). In Europe, overweight and obesity are already a common phenomenon, with 60% of adults are already obesity or overweight, and childhood obesity rates approaching 30% (World Health Organization, 2022). According to the Chinese Residents Nutrition and Chronic Disease Status Report (State Council, 2020), more than half of Chinese adults were overweight or obesity, and the rate of overweight or obesity among children and adolescents was close to 20%, and the rate of overweight or obesity among children under 6 years old had also reached 10% (State Council, 2020). Obesity is a threat to human health and can induce a variety of chronic diseases (Dye et al., 2017). Moreover, studies have found that cognitive functions, including memory, execution, language, understanding, judgment and other abilities, also decline with the occurrence of obesity (Li et al., 2008). It is expected that with the further epidemic of obesity, there will be more people with cognitive disabilities worldwide. Therefore, it is important to investigate the mechanism of obesity-related cognitive dysfunction (ORCD) to protect human health. Obesity can reduce neurogenesis, decrease synaptic plasticity and induce oxidative stress and inflammation, and changes in these physiological mechanisms can cause cognitive decline (Liu et al., 2015; Petronilho et al., 2016; Bracke et al., 2019; Lewis et al., 2019). In contrast, insulin-like growth factors-1 (IGF-1) can inhibit these physiological changes caused by obesity, and exerting a cognitive protective function (Della Sala et al., 2016; Carlson and Saatman, 2018).

Exercise, as a non-drug therapy, has been proved to have a good therapeutic effect on a variety of diseases in clinical studies or animal experiments. Further studies have shown that exercise can improve neurogenesis, synaptic plasticity, and reduce oxidative stress and inflammation by promoting IGF-1 expression (Cao et al., 2020; Kang et al., 2020; Lin et al., 2020; Li et al., 2022). This suggests that exercise can also play an excellent therapeutic effect in ORCD, alleviating cognitive decline. In this review, we summarized the effects of obesity and IGF-1 on cognitive function, and discusses the mechanism by which exercise improved ORCD through regulating IGF-1. This study lays a foundation for further research on the mechanism and treatment of ORCD.

## 2. The association between obesity and cognitive function

### 2.1. Effects of obesity on cognitive function in animals

There have been many studies confirming that obesity impairs cognitive function. In animal studies, many experiments have been conducted to evaluate the effect of obesity on cognitive function by inducing obesity in animals through High-fat diet (HFD) feeding and observing the performance of the animals in relevant cognitive tests. Liu et al. (2014) successfully induced obesity in mice by feeding them a HFD for 16 weeks, and observed a decline in the ability to learn and remember, and cognitive dysfunction in the experiments in the obese mice. The cognitive function of animals can be studied by different cognitive test protocols, and the Morris water maze (MWM) experiment is a cognitive test protocol that is commonly used to test the learning and memory function of animals. Research by Cheng et al. (2016), obesity was also induced by a 16-week high-fat diet, and it was observed in the MWM experiment that obese mice spent significantly less time on the target quadrant than the control group, and the escape latency of the obese mice was also significantly prolonged, which suggests that obesity reduces the spatial learning and memory abilities of the mice, and impairs their cognitive functions. The Y-maze experiment is also an experimental method to test the spatial learning and memory ability of animals. Gladding et al. (2018) showed that obese mice not only performed poorly in the MWM experiment, but also spent less time exploring the novel arm and took longer to reach the novel arm in the Y-maze, which proved once again that obesity impaired the spatial memory ability of the mice, leading to hippocampus-dependent cognitive impairment. The New object recognition (NOR) task is a test protocol for observing the recognition memory ability of animals. In the NOR experiment, obese mice and rats showed a significant decrease in the ability to recognize new objects compared to healthy controls, suggesting that obesity reduces recognition memory ability and induces cognitive deficits (Wang et al., 2015; Lewis et al., 2019). Obesity not only causes cognitive deficits in otherwise healthy individuals, but also aggravates the effects of some pathological states on cognitive function. For example, a model of postoperative cognitive dysfunction in aged mice showed hippocampus-dependent memory loss after surgery, and obesity significantly worsened this cognitive dysfunction in mice (Zhao et al., 2020).

### 2.2. Effects of obesity on cognitive function in humans

Currently, many studies have focused on the correlation between obesity and cognitive function, and most of the evidence suggested that obesity can cause different degrees of decline in human cognitive ability. For example, in special groups (school-age children, adolescents, and the elderly), it has been demonstrated that obesity was significantly associated with cognitive function through relevant cognitive function tests. Obesity can impair children’s executive function and significantly reduce visuospatial organization ability and general mental ability (Li et al., 2008; Ronan et al., 2020). Obese and overweight older adults showed poor performance in overall cognitive function, verbal fluency, immediate and delayed logical memory, mental speed and intelligence (Benito-León et al., 2013). Clinical studies on adults have shown cognitive decline in obese adults with major depression, especially in working memory, processing speed and executive function (Hidese et al., 2018). Studies in the middle-aged population suggested that obesity reduced executive function (Nazmus Sakib et al., 2022). The above studies have well demonstrated that obesity was correlated with cognitive decline. Cognitive function in humans was affected by obesity from childhood to the elderly, and that executive function might be more susceptible to be inhibited in cognitive domains affected by obesity. However, some studies have shown that elderly people with higher body mass index have a lower probability of developing mild cognitive impairment (Zhao et al., 2019). The differential results in the study of obesity cognitive impairment may be related to indicators for evaluating obesity.

Due to the body mass index (BMI) cannot accurately reflect the degree of obesity, it is necessary to establish other obesity indicators in studying the relationship between obesity and cognitive function. A cross-sectional study reported a significant correlation between waist to hip ratio (WHR) and executive function in young and middle-aged individuals, indicating a correlation between weight gain and cognitive decline in young individuals (Hartanto et al., 2019). Lin (2022) also confirmed that men and women with larger WHR performed worse on mini mental state examination (MMSE) tests and showed a decrease in cognitive ability. In addition to BMI and WHR, the ratio of fat mass (FM) to fat free mass (FFM) not only represented obesity, but also distinguished between muscle mass and fat mass. A recent cross-sectional study evaluating cognitive impairment in elderly people (≥60 years old) through MMSE showed that the risk of cognitive impairment in elderly people increases linearly with the increase of FM/FFM ratio (Ma et al., 2021). In summary, human studies have shown that obesity can lead to cognitive dysfunction, and inconsistent results in human studies might be due to different obesity indicators or cognitive function testing protocols chosen.

## 3. Mechanism of cognitive dysfunction caused by obesity

### 3.1. Obesity and neurogenesis

Neurogenesis is the process by which neural stem cells and neuronal progenitor cells give rise to neurons and involves the proliferation, migration, differentiation, survival and integration of new neurons into existing circuits. Neurogenesis is closely linked to cognitive function, for example, rats exposed to isoflurane showed inhibition of neurogenesis in the sublateral ventricular zone and subgranular layer, a significant reduction in neural stem cell proliferation, and learning memory impairments (Wang et al., 2018). It has also been shown that the learning memory ability of mice can be reduced or enhanced after inhibiting or promoting neurogenesis by drugs (Ren et al., 2009). As an indispensable physiological process in normal brain activity, the impact of neurogenesis on cognitive function is also evident, and maintaining normal neurogenesis helps to protect cognitive function.

Obesity can impair normal neurogenesis. In leptin-deficient obese mice, both phosphorylated histone H3 level (a marker of cell proliferation in the hippocampal dentate gyrus (DG)) and the number of bicortin-positive cells (a marker of neuronal differentiation) were significantly decreased, indicating that neurogenesis was inhibited in obese mice (Bracke et al., 2019). Furthermore, in HFD-induced obese mice, the number of brdu-positive cells and the number of new and/or immature neurons in the DG of the mice hippocampus was reduced (Park et al., 2010; Robison et al., 2020). Thus, these studies suggested that obesity reduced neuroproliferation and inhibited neurogenesis. Considering the link between neurogenesis and cognitive function, obesity might impair cognitive function by reducing neurogenesis. This is supported by the study of Tanokashira et al. (2018) who successfully induced obesity in mice by HFD and observed impaired neurogenesis and hippocampus-dependent cognitive decline in obese mice. Thus, diminished neurogenesis in the brain may be one of the mechanisms of ORCD.

### 3.2. Obesity and synaptic plasticity

Synaptic plasticity refers to the ability to alter the strength of synaptic connections in neuronal networks, and good synaptic plasticity is fundamental to maintaining normal cognitive function (Magee and Grienberger, 2020). It has been reported that in the mouse model with reduced field excitatory synapse potentials (fEPSPs) induced by drugs, synaptic plasticity in mice was damaged, and its performance in MWM and NOR tasks was worse, indicating that inhibiting synaptic plasticity would damage the ability of recognition and spatial memory in mice (Guo et al., 2021). Radiation is also a method of inhibiting synaptic plasticity, and animal studies have shown that mice exhibit cognitive deficits after receiving intracranial irradiation at a dose rate of 3 Gy/min for 3 months, as well as significant reductions in spiking discharges and excitatory synaptic inputs, and significant enhancement of inhibitory inputs to hippocampal CA1 pyramidal neurons, and reduced expression of the synaptic plasticity marker, suggesting that radiation may induce cognitive deficits by inhibiting synaptic plasticity (Wu et al., 2022).

In studies of ORCD, it is also found that obesity impairs synaptic plasticity. For example, the expression of synaptic markers in obese mice was decreased and the long-term potentiation (LTP) in hippocampal CA1 region was significantly weakened, indicating synaptic plasticity was impaired (Liu et al., 2015; Nam et al., 2017). In addition, obesity reduced dendrite spine density in vertebral neurons and inhibited the expression of synaptic markers in the prefrontal cortex and limbic cortex, and these changes were associated with decreased performance in cognitive tasks of rats (Bocarsly et al., 2015). High frequency stimulation is a electrophysiology method to induce fEPSPs and evaluate LTP. Studies have shown that high-frequency stimulation does not significantly enhance fEPSEs in the hippocampus of mice fed with short-term HFD, indicating that short-term HFD damages LTP in the hippocampus of mice (Wang et al., 2020). The above studies suggest that obesity inhibits LTP, reduces dendritic spine density, and impairs normal synaptic plasticity, thereby possibly contributing to cognitive deficits.

### 3.3. Obesity and oxidative stress

Oxidative stress refers to the excessive production of oxygen free radicals that cannot be timely removed by the antioxidant system, resulting in an increase in oxygen free radicals and attacking the body or cells and leading to oxidative damage. Previous studies have shown that cognitive decline is related to systemic oxidative stress (Berr et al., 2000). Animal studies have also shown that in the oxidative stress induced by aging in rats, long-term administration of metformin can reduce the level of malondialdehyde (MDA, one commonly used marker of oxidative damage) in the rat hippocampus, enhance the total antioxidant capacity and passive avoidance learning ability, indicating that the age-dependent oxidative stress and cognitive function of rats have been improved (Gorgich et al., 2021). In the Chinese elderly, higher green tea intake can improve antioxidant capacity, reduce AD-related pathological changes and protect memory and executive function (Zhang et al., 2022). It can be seen that cognitive function will be inhibited by oxidative stress, and cognitive function can be protected by improving the body’s antioxidant capacity.

Obesity had a strong correlation with oxidative stress. Oxidative damage was induced in septic rats, and obesity aggravated septic induced peripheral organ damage (Petronilho et al., 2016). It has also been reported in clinical studies that the levels of MDA and protein carbonylation were reduced in obese women after bariatric surgery (Horn et al., 2017). Both animal experiments and human studies have shown that obesity can cause or aggravate oxidative stress, and oxidative stress has a certain correlation with cognitive function, so oxidative stress may be one of the reasons for ORCD.

### 3.4. Obesity and chronic inflammation

Inflammation is a protective response of the body to harmful stimuli. When the body reacts with inflammation, the levels of inflammatory factors such as tumor necrosis factor α (TNF-α), interleukin-1β (IL-1β), and IL-6 is increased. Recently, inflammation has been linked to cognitive dysfunction. For instance, pro-inflammatory diets can induce serum inflammation and lead to cognitive dysfunction in postmenopausal women, while anti-inflammatory diets can reduce inflammatory responses and improve cognitive function (Skoczek-Rubińska et al., 2021). In addition, inflammation has been reported to mediate cognitive dysfunction in obese/overweight patients with major depression (Lan et al., 2022). This suggests that inflammation can lead to cognitive dysfunction.

Chronic inflammation was one of the symptoms of obesity. Obesity induced the occurrence and development of chronic inflammation through various mechanisms such as hypertrophic necrosis of adipocytes, hypoxia, oxidative stress, endoplasmic reticulum stress, impaired peroxisome proliferator-activated receptors, inflammatory vesicle activation and Toll-like receptor activation (Artemniak-Wojtowicz et al., 2020). Both human and animal studies have shown that obesity induced an inflammatory response *in vivo*, with increased levels of inflammatory factors (such as IL-1β, IL-6, and TNF-α) (Lewis et al., 2019; Lan et al., 2022). As inflammation was associated with cognitive function, chronic inflammation might also play an important role in ORCD, which needs to be studied in depth.

## 4. IGF-1 and cognitive function

IGF-1 is a polypeptide homologous to insulin, which can promote cell growth, cell differentiation and bone synthesis and metabolism. IGF-1 in the body was mainly produced in the liver and released into the blood. IGF-1 in plasma can prolong the half-life and increase bioavailability by binding with IGF binding protein (IGFBP), and can be actively transported from plasma to the central nervous system (CNS) (Labandeira-Garcia et al., 2017) through the choroidal membrane and across the blood–brain barrier. IGF-1 was then isolated from IGFBP through an enzymatic process and bound to the IGF-1 receptor (IGF-1R) with high affinity (Orrù et al., 2017), activating the downstream signaling pathway to perform corresponding functions (Al-Samerria and Radovick, 2021). The cortex, hippocampus, cerebellum, hypothalamus, subventricular zone and dentate gyrus in the CNS also produced IGF-1, which together with peripheral IGF-1 regulated neuronal growth, development, metabolism and neuroplasticity (Dyer et al., 2016).

### 4.1. Correlation of cognitive function with IGF-1

Current evidence suggests that the level of IGF-1 is positively correlated with cognitive ability in humans. In a prospective population study based on older adults, the level of IGF-1 was found to be correlated with cognitive ability (Dik et al., 2003). In this study, it was observed that the elderly with the lowest serum IGF-1 level (<9.4 nmol/L) showed a significant decrease in information processing speed. Moreover, serum IGF-1 level was positively correlated with working memory, selective attention, and executive control function in a healthy population of older adults using cognitive tests including MMSE, the wiring test A-B, the Ruff’s test for selective attention, and the alphanumeric ordering test (Bellar et al., 2011). One neuropsychological test found that in patients with type 2 diabetes combined with mild cognitive impairment, the serum IGF-1/IGFBP-3 concentration ratio was reduced, and the executive function of patients was significantly decreased (Huang et al., 2015). This was also confirmed by Calvo et al. (2013) who found that among patients with mild cognitive impairment, patients with higher serum IGF-1 level had better cognitive performance in learning and memory tests. In addition, an association between cognitive function and IGF-1 had also been found in neurodegenerative diseases with cognitive decline. For example, in patients with early Parkinson’s disease (PD), low serum IGF-1 was associated with poor performance on cognitive tasks that assess executive function, attention, and verbal memory (Picillo et al., 2017). Another study of MMSE and linear logistic regression analysis also showed that poor cognitive performance in PD patients was correlated with lower plasma IGF-1 level (Ma et al., 2015). Moreover, among patients with multiple sclerosis, patients with cognitive impairment had significantly lower level of IGF-1 than those with normal cognition (Nageeb et al., 2018). In conclusion, these studies indicate a strong positive association between IGF-1 and cognitive function, and IGF-1 may serve as a marker for predicting cognitive function in humans.

### 4.2. Animal studies: the regulation of IGF-1 on cognitive function

In animal studies, IGF-1 has been shown to regulate cognitive function. For example, sevoflurane anesthesia can induce the decrease of serum IGF-1 level in mice, thus the performance of mice in MWM was worse, indicating that the spatial learning and memory ability of mice is impaired (Jiang et al., 2017). This is due to that sevoflurane reduced serum IGF-1 level, leading to a decrease in IGF-1 entering the CNS through the blood–brain barrier, which in turn might reduce β-amyloid clearance from the brain and hyperphosphorylated tau proteins, thereby impairing cognitive function. The liver was the main site of IGF-1 secretion, and mice with liver-specific targeted IGF-1 gene disruption had reduced serum IGF-1 level and performed poorly in the rotational assay and MWM, indicating poor spatial learning and memory abilities and cognitive dysfunction (Trejo et al., 2007). This was also confirmed by Pharaoh et al. (2020) they found that circulating IGF-1 deficiency in male mice significantly impaired hippocampus-dependent spatial and reversal learning. From the above studies, it was clear that decreased IGF-1 level impaired spatial learning and memory abilities and reduced cognitive function in mice.

Increased level of IGF-1 may protect cognitive function. Subcutaneous injection of IGF-1 into rats during the early stage of sepsis (within 6 h) could reduce cell apoptosis, improve the performance of septic rats in MWM and passive avoidance experiments, and protect their memory and spatial learning abilities (Yang et al., 2019). Similarly, IGF-1 gene treatment in rats with traumatic brain injury reduced oxidative stress generated in the prefrontal cortex, motor cortex and hippocampus and improved the performance of rats in the Y-maze, promoting their working memory capacity and preventing cognitive deficits caused by traumatic brain injury (Montivero et al., 2021). In the rat model of cognitive dysfunction induced by sevoflurane anesthesia, intravenous injection of IGF-1 increased the level of IGF-1 protein in plasma, hippocampus and cortex of rats, thereby enhancing the performance of rats in MWM and inhibiting cognitive dysfunction (Jiang et al., 2017; Xie et al., 2021). In addition, in animal models of ischemic stroke and aging, increased level of IGF-1 in animals through exogenous injection or targeted gene therapy could also improve the performance of animals in MWM or antistatic passive avoidance tests, reduce spatial memory deficits and learning impairment (Hu et al., 2016; Yang et al., 2020). These studies suggest that increasing IGF-1 level in animals facilitates learning and memory abilities, thereby improving cognitive deficits.

## 5. The effect of exercise on cognitive dysfunction in obesity

### 5.1. Effects of aerobic exercise on cognitive dysfunction in obesity

Currently, many studies support the role of exercise in improving ORCD. Animal studies have found that cognitive dysfunction in HFD mice is alleviated after treadmill training. Specifically, 12 weeks of treadmill exercise improved the performance of obese mice in the Y-maze, showing that exercise promoted short-term memory and spatial memory in obese mice (Kim et al., 2016). This was also demonstrated in the study of Park et al. (2019) who found that 12 weeks of treadmill exercise effectively improved the performance of obese mice in the MWM and inhibited ORCD. The above studies show that exercise plays a positive role in improving ORCD. Additionally, animal studies have shown that exercise combined with dietary interventions can work together to improve ORCD (Woo et al., 2013).

However, when performing aerobic exercise, the effect of different intensities on ORCD is still controversial. Studies have found that 8 weeks of low-intensity (40–45% maximum speed) or high-intensity (75–80% Maximum speed) treadmill exercise can improve the performance of obese mice in the Y maze, and there is no significant difference between the two exercise intensities on cognitive function (Bae, 2020). However, Kim et al. (2020) showed that only high-intensity aerobic exercise performed better in the MWM in obese mice compared with low- and moderate-intensity aerobic exercise. The differential effects of exercise intensity on cognitive function may be related to cognitive testing protocols and further research is needed to confirm this.

A positive effect of combining exercise and dietary interventions on improving ORCD has also been found in human studies (Peven et al., 2020). Specifically, a 12-month energy-restricted diet combined with moderate- or high-intensity aerobic exercise improved cognitive function in obese adults, and high-intensity exercise combined with dietary interventions improved cognitive function best. Similarly, in a study on exercise intensity, Inoue et al. (2020) showed that moderate-intensity and high-intensity aerobic exercise 3 times per week for 6 weeks improved executive function in obese adults. At the same time, we also noted that diet structure may have an impact on exercise effectiveness. In the natural state, protein intake may increase during exercise in humans, and high-protein diets have been shown to promote IGF-1 expression (Gulick et al., 2020). Therefore, cognitive function in the obese state may be affected by both exercise and dietary interventions, suggesting that we should strictly control dietary structure and reduce the influence of irrelevant factors in human studies.

### 5.2. Effects of resistance exercise on cognitive dysfunction in obesity

In addition to aerobic exercise, resistance exercise is also an effective way to improve ORCD. For example, by performing resistance exercise (ladder climbing) for 8 weeks, the performance of obese mice in the Y-maze was significantly enhanced, suggesting that resistance exercise improves cognitive function in obese mice (Lim et al., 2022). In addition to studying the impact of exercise intensity on cognitive function, some scholars have compared the impact of different exercise methods on ORCD. For example, Maryam and Ali let obese rats perform aerobic exercise and resistance exercise respectively, and observed the effects of these two kinds of exercise on cognitive function (Maryam and Ali, 2023). The results showed that both kinds of exercise can improve the cognitive dysfunction of obese rats, and there was no significant difference in the degree of improvement in cognitive function. Recent studies have looked at the role of combined aerobic and resistance exercise interventions on ORCD. As demonstrated by Lee et al., a 12-week combined aerobic and resistance exercise training can improve short-term memory in obese rats (Lee et al., 2022).

In human studies, it has also been found that after 12 weeks of resistance training (60–70% 1RM), the scores of elderly obese women in the Montreal cognitive assessment can be improved, indicating that resistance training improves cognitive function (De Camargo Smolarek et al., 2019). Some scholars have also paid attention to the impact of resistance exercise of different intensity on ORCD. A recent study showed that the executive function of obese elderly people was significantly improved after a single resistance exercise, and there was no significant difference in the impact of resistance exercise of 50% 1RM and 70% 1RM on cognitive function (De Almeida et al., 2021).

## 6. Effects of exercise on IGF-1 level

### 6.1. Regulation of IGF-1 by aerobic exercise

Early studies have shown that aerobic exercise promotes the expression of IGF-1 in the body, for example, Cetinkaya et al. subjected adolescent rats to a total of 12 weeks of aerobic exercise, 6 weeks of treadmill exercise and 6 weeks of rollerblading, and they found that the levels of IGF-1 in the serum and the hippocampus of the rats were significantly elevated after the exercise (Cetinkaya et al., 2013). And subsequent studies have found gender differences in the effects of aerobic exercise on IGF-1. For instance, animal studies have shown that the levels of IGF-1 in the hippocampus of male and female rats were significantly elevated after exercise only in male rats, and the changes in the levels of IGF-1 in female rats were not significant (Uysal et al., 2017). Similarly, this result has been experimentally demonstrated in human studies, where 12 weeks of aerobic exercise did not significantly alter growth hormone and IGF-1 levels in older women in a study by Seo et al. (2010). However, a study in older men found that serum IGF-1 levels increased significantly after aerobic exercise (Arazi et al., 2021). Regarding the gender difference in the effect of exercise on IGF-1, which may be related to sex hormones, a previous study reported that intraperitoneal injection of estradiol in rats increased the expression of IGF-1 in the CAI region of the hippocampus (Wang and Xu, 2010). Whereas, with age, estrogen levels decrease, which may then affect the promotion of IGF-1 by exercise in older women.

The IGF-1 level in the body decreased with aging, and in the elderly with mild cognitive impairment (MCI), aerobic exercise intervention lasting for 18 months can significantly increase the serum IGF-1 level and improve cognitive ability (Xie and Wu, 2017). In addition, the promoting effect of aerobic exercise on IGF-1 might be different in different pathological states. For example, acute aerobic exercise increased circulating IGF-1 level in Alzheimer’s disease (AD) patients, but had no significant effect on elderly people without dementia. This might be due to lower circulating IGF-1 level in AD patients, and the stimulative effect of exercise would be more pronounced (Stein et al., 2021). Other studies have reported that the effect of aerobic exercise on promoting IGF-1 expression was not as obvious as that of resistance exercise (Shanazari et al., 2019).

### 6.2. Regulation of IGF-1 by resistance exercise

Resistance exercise is a type of exercise that allows muscles to fight resistance, and many studies have shown that resistance exercise promotes IGF-1 expression. For example, low-intensity resistance exercise twice daily for 2 weeks combined with muscular venous blood flow restriction could increase circulating IGF-1 concentration (Abe et al., 2005). However, there were gender differences in the effects of resistance exercise on IGF-1. Studies have shown that 6 weeks of whole-body resistance training had no significant effect on serum IGF-1 level in women (Ansari Kolachahi et al., 2020). In contrast, there was a significant increase in serum IGF-1 in young men after only one acute resistance exercise (Tsai et al., 2014). And this study also observed that high-intensity (80% 1RM) resistance exercise had better effects than moderate intensity (50% 1RM) resistance exercise, with a more significant increase in IGF-1 level. Approximately 20 min after a single acute resistance exercise, the increased serum IGF-1 level was significantly reduced, suggesting that long-term high-intensity resistance exercise might have a more significant promoting effect on IGF-1 abundance (Tsai et al., 2014).

The effects of resistance exercise on IGF-1 might also be related to exercise duration and age, as it has been shown that resistance exercise could increase IGF-1 levels in the body, but this effect was more significant in subjects over 60 years of age with training duration ≤16 weeks (Jiang et al., 2020). These studies indicated that resistance exercise had the effect of promoting IGF-1 expression, but this effect might be more significant in male, and the effect was more pronounced with older age and higher load intensity. This might be because the level of IGF-1 decreased with age, making the training effect more prominent. It should be noted that the selection of high-intensity resistance exercise in the elderly needed to pay attention to the elderly’s physical bearing capacity. In addition, training time also affected the effect of resistance exercise. It took a long training period to steadily improve the IGF-1 level of the body, but too long a training period might cause the body to produce adaptability and reduce the training effect.

## 7. The role of IGF-1 in exercise to improve cognitive dysfunction in obesity

### 7.1. The role of IGF-1 in exercise improvement neurogenesis

IGF-1 has been found to promote hippocampal neurogenesis, such that brain-injured mice with central infusion of IGF-1 showed a significant increase in the density of immature hippocampal neurons and attenuated brain injury-induced motor and cognitive dysfunction (Carlson and Saatman, 2018). In addition, IGF-1 overexpression increased neuronal proliferation and differentiation in DG in transgenic elderly mice, and led to better performance in MWM and anti-hypertensive tests, suggesting that IGF-1 can improve cognitive function in elderly mice by stimulating hippocampal neurogenesis (Hu et al., 2016). And the effect of IGF-1 was more pronounced in the hippocampus where neurogenesis was inhibited, as one study reported that IGF-1 overexpression increased neonatal neurons in brain-injured mice, whereas in control uninjured mice, IGF-1 overexpression did not significantly increase neonatal neuronal density (Carlson et al., 2014). These studies suggest that IGF-1 has a role in promoting neurogenesis, but the effect may be influenced by pathological status.

Exercise can promote neurogenesis, and this effect is closely related to IGF-1. Studies have shown that the 4-week voluntary exercise of mice up-regulates the expression of IGF-1 and promotes hippocampal neurogenesis, and this effect of exercise is mediated by IGF-1, as blocking the effect of IGF-1 by intraperitoneal injection of IGF-1R inhibitor significantly inhibited learning memory ability and hippocampal neurogenesis in mice (Cao et al., 2020). The promotion effect of exercise on IGF-1 abundance might also be regionally specific. According to the study of Yu et al., 15 days of voluntary exercise increased the expression of IGF-1 and promoted hippocampal neurogenesis in healthy adult mice compared with the non-exercise group, but the expression of IGF-1 only increased in the DG region (Yu et al., 2014). In addition, it has been shown that 6 weeks of resistance exercise (ladder climbing) can activate the IGF-1 downstream signaling pathways external-signal regulated kinase 1/2 (ERK1/2) and glycogen synthase kinase 3β (GSK-3β) in the DG region and enhance cell proliferation in the DG, improving lipopolysaccharide-induced spatial learning deficits in rats (Kelty et al., 2019). It has also been shown that exercise training promoted neural progenitor cell proliferation by activating the IGF-1/protein kinase B (protein kinase B, PKB, or Akt) pathway (Zheng et al., 2014). This suggests that exercise promotes neurogenesis by enhancing IGF-1 expression and activating Akt, ERK1/2, and GSK-3β signaling pathways.

### 7.2. The role of IGF-1 in exercise to improve synaptic plasticity

The change of IGF-1 level also affects hippocampal synaptic plasticity. Overexpression of IGF-1 has been found to enhance dendritic ramification of immature hippocampal neurons in mice with traumatic brain injury (Carlson et al., 2014) and improve dendrite spinous density in adult Cdkl5^−/y^ mice (Della Sala et al., 2016). This suggests that IGF-1 strengthens synaptic connections. IGF-1 can also regulate the intensity of synaptic transmission. For example, serum IGF-1 deficiency can damage LTP in the hippocampus, and hippocampus LTP impairment was associated with decreased density of glutamatergic synapses, which caused imbalance of glutaminergic/GABaergic synaptic ratio in the brain region, and ultimately caused cognitive dysfunction. Systemic administration of IGF-I improved MWM performance and synaptic defects of serum IGF-I deficient mice, and normalized hippocampal glutaminergic synaptic density (Trejo et al., 2007). Impairment of hippocampal LTP was associated with a decrease in calmodulin-dependent protein kinase II (CaMK) activity (Hu et al., 2016). After blocking the CaMK II signaling pathway, mice performed worse in MWM, while transgenic mice overexpressing IGF-1 had higher levels of CaMK II phosphorylation and performed better in MWM (Hu et al., 2016). The above studies indicate that IGF-1 improves synaptic plasticity by promoting the growth of synaptic connections and maintaining the strength of normal synaptic transmission.

Exercise improves synaptic plasticity by promoting IGF-1 expression. For example, voluntary exercise activated IGF-1/CaMK II signaling pathway, increased the release of synaptic protein 1, and improved hippocampal synaptic plasticity by promoting the expression of IGF-1 in rat hippocampus (Ding et al., 2006). Different types of exercise can improve synaptic plasticity by activating different signaling pathways. Both aerobic exercise and resistance exercise can inhibit mild stress-induced apoptosis of hippocampal neurons, increase the amplitude of population peak potential and the slope of fEPSP, and enhance LTP in hippocampal CA1 region (Kang et al., 2020).

However, the study found that the two types of exercise activate different molecular pathways. Aerobic exercise, while increasing IGF-1 protein expression, appeared to be more activating of the peroxisome proliferator-activated receptor γ coactivator 1α (PGC-1α)/Estrogen Related Receptor α (ERRα)/Fibronectin type III domain-containing protein 5 (FDNC5) signaling pathway, while resistance exercise tended to upregulate the IGF-1/IGF-1R/Akt/mammalian target of rapamycin (mTOR) signaling pathway. These two signaling pathways have different roles in improving synaptic plasticity, with the IGF-1/IGF-1R/Akt/mTOR signaling pathway being involved in the regulation of protein synthesis, while the PGC-1α/ERRα/FDNC5 signaling pathway plays a more important role in neuronal cell survival, differentiation and plasticity (Cho et al., 2013). Exercise can also act in combination with other intervention modalities to enhance hippocampal plasticity. For example, 3 weeks of environmental enrichment combined with comprehensive exercise (aerobic exercise combined with resistance exercise) increased serum IGF-1 level in rats, and the number of cells in the DG and CA1 regions was significantly increased, which might promote hippocampal plasticity (Rostami et al., 2021).

### 7.3. The role of IGF-1 in exercise ameliorating oxidative stress

Currently, some studies have confirmed that IGF-1 has antioxidant effects. In the brain tissue of IGF-1-deficient mice, MDA increased and gene expression of related antioxidant enzymes decreased, suggesting that IGF-1 deficiency caused oxidative stress in the mice brain, but low-dose IGF-1 treatment restored all these indicators to normal levels in IGF-1-deficient mice (Puche et al., 2016). IGF-1 can also exert antioxidant effects in different physiological states. For example, in animal models of aging or brain injury, oxidative damage in the brain can be attenuated by IGF-1 infusion (García-Fernández et al., 2008; Montivero et al., 2021). These findings suggest that IGF-1 is an important factor affecting oxidative stress, and IGF-1 deficiency exacerbated oxidative stress. And in some specific physiological states, oxidative stress can be attenuated by exogenous supplementation of IGF-1, which might be one of the mechanisms by which IGF-1 protects cognitive function.

Moderate aerobic exercise can protect the brain’s health by improving its antioxidant capacity and enhancing its resistance to oxidative stress (Simioni et al., 2018). Some studies further demonstrated that exercise can improve oxidative stress by enhancing IGF-1 expression. For example, the proliferation of cells in the hippocampal DG region of stress induced-mice was decreased and cognitive function of mice was declined, while autonomic exercise can reduce stress-induced proliferation injury and cognitive dysfunction by promoting the expression of IGF-1 in the cerebral cortex and liver and enhancing the activity of glutathione S-transferase in the brain (Nakajima et al., 2010). In addition, 4 weeks of aerobic exercise also promoted the expression of IGF-1 in the hippocampus of aged rats and prevented an increase in age-related reactive oxygen species and lipid peroxidation levels, which had a positive effect on cognitive function in aged rats (Vanzella et al., 2017). Further research suggested that different types of exercise might have different effects on oxidative stress. A recent study demonstrated that aerobic exercise, resistance exercise, whole body vibration and electrical stimulation can increase skeletal muscle mass, exercise capacity, metabolic indices and protein synthesis, and inhibit oxidative stress and apoptosis by activating IGF-1 pathway, among which resistance exercise had the best effect (Li et al., 2022).

### 7.4. The role of IGF-1 in exercise improvement on inflammation

IGF-1 also performs anti-inflammatory functions *in vivo*. In mice with neuroinflammation and cognitive impairment induced by sleep deprivation, intravenous IGF-1 injection activated the PI3K/Akt/GSK-3β signaling pathway to reduce neuroinflammation and prevent cognitive decline (Wan et al., 2022). Further studies showed that IGF-1 exerted anti-inflammatory functions in elderly female rats by promoting the polarization of microglia/macrophages toward an anti-inflammatory phenotype, down-regulating genes related to inflammation and microglia response (Falomir-Lockhart et al., 2022). In addition, some studies demonstrated that IGF-1 can also play a protective role in the brain of stroke rats, and the lateral ventricular infusion of IGF-1 in post-stroke female rats can reduce the volume of cerebral infarction and inhibit the expression of pro-inflammatory factors (Bake et al., 2014). This study also found that IGF-1 treatment inhibited levels of inflammatory cytokines such as TNF-α, IL-1β, and IL-6 at 4 h after stroke, while only IL-6 and IL-13 continued to be inhibited at 24 h after stroke, as did chemokines GRO-KC and CCL2. This suggests that IGF-1 has a sustained inhibitory effect on specific inflammatory cytokines.

Early studies have found that exercise can reduce inflammation and increase IGF-1 levels (Ben Ounis et al., 2010). Exercise can improve the anti-inflammatory environment by reducing adipose tissue and inhibiting inflammatory release (Calcaterra et al., 2022). IGF-1 has also been found to mediate the anti-inflammatory function of exercise. Lin et al. reported that 8-week swimming inhibited hippocampal inflammation and apoptosis induced by D-galactose in male rats by enhancing IGF-1R/PI3K/Akt signaling pathway (Lin et al., 2020). In this study, Lin also found that exercise played an anti-inflammatory role in the hippocampus of D-galactose-induced aging rats, while exercise instead promoted hippocampal inflammation in non-aging rats. This suggests that exercise might have anti-inflammatory or proinflammatory effects under different physiological conditions, which need further study.

## 8. Conclusion

**Figure 1:**
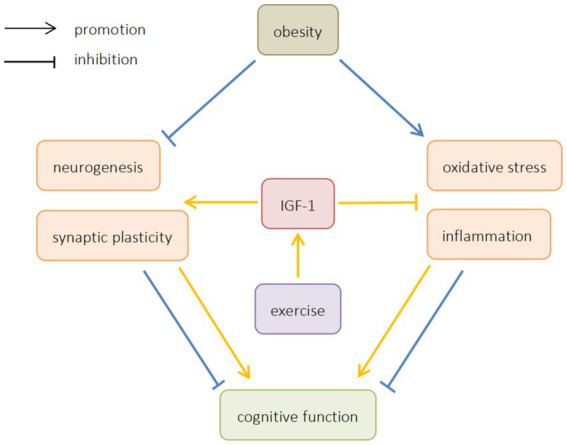
The effects of obesity and exercise on cognitive function. IGF-1, insulin-like growth factors-1.

This article reviews the mechanism of ORCD, and analyzes the possibility of exercise improving ORCD by regulating IGF-1. It is believed that exercise can increase the expression of IGF-1, promote neurogenesis and synaptic plasticity, inhibit oxidative stress and inflammation, and improve ORCD (Figure 1). In light of the above studies, exercise can be considered as a possible strategy to prevent cognitive impairment in obese patients. However, most of the existing studies are animal studies, and there is still a lack of relevant human studies to provide direct evidence that exercise can improve cognitive function by regulating IGF-1 in obese patients. At the same time, the current research on how exercise can improve oxidative stress and inflammation by regulating IGF-1 is not enough, and further research is needed. Therefore, further human studies should be carried out in the future to explore the effect of exercise on IGF-1 and ORCD.

## Author contributions

YH drafted the manuscript. ZP and XG helped to complete the manuscript. HW, QW, and YD collected the data. NJ revised the manuscript. All authors have read and approved the final manuscript.

## Funding

This work was supported by the National Natural Science Foundation of China (31370021).

## Conflict of interest

The authors declare that the research was conducted in the absence of any commercial or financial relationships that could be construed as a potential conflict of interest.

## Publisher’s note

All claims expressed in this article are solely those of the authors and do not necessarily represent those of their affiliated organizations, or those of the publisher, the editors and the reviewers. Any product that may be evaluated in this article, or claim that may be made by its manufacturer, is not guaranteed or endorsed by the publisher.
